# MAVS maintains mitochondrial homeostasis via autophagy

**DOI:** 10.1038/celldisc.2016.24

**Published:** 2016-08-16

**Authors:** Xiaofeng Sun, Liwei Sun, Yuanyuan Zhao, Ying Li, Wei Lin, Dahua Chen, Qinmiao Sun

**Affiliations:** 1 State Key Laboratory of Membrane Biology, Institute of Zoology, Chinese Academy of Sciences, Beijing, China; 2 State Key Laboratory of Membrane Biology, Tsinghua University-Peking University Joint Center for Life Sciences, School of Life Sciences, Tsinghua University, Beijing, China; 3 State Key Laboratory of Stem Cell and Reproductive Biology, Institute of Zoology, Chinese Academy of Sciences, Beijing, China

**Keywords:** autophagy, MAVS, mitochondrial homeostasis

## Abstract

Mitochondrial antiviral signalling protein (MAVS) acts as a critical adaptor protein to transduce antiviral signalling by physically interacting with activated RIG-I and MDA5 receptors. MAVS executes its functions at the outer membrane of mitochondria to regulate downstream antiviral signalling, indicating that the mitochondria provides a functional platform for innate antiviral signalling transduction. However, little is known about whether and how MAVS-mediated antiviral signalling contributes to mitochondrial homeostasis. Here we show that the activation of MAVS is sufficient to induce autophagic signalling, which may mediate the turnover of the damaged mitochondria. Importantly, we find MAVS directly interacts with LC3 through its LC3-binding motif ‘YxxI’, suggesting that MAVS might act as an autophagy receptor to mediate mitochondrial turnover upon excessive activation of RLR signalling. Furthermore, we provide evidence that both MAVS self-aggregation and its interaction with TRAF2/6 proteins are important for MAVS-mediated mitochondrial turnover. Collectively, our findings suggest that MAVS acts as a potential receptor for mitochondria-associated autophagic signalling to maintain mitochondrial homeostasis.

## Introduction

Upon viral infection, the host innate immune system employs several pattern recognition receptors, including membrane-bound Toll-like receptors and cytosolic receptors, such as retinoic-acid-inducible gene-I (RIG-I)-like receptors (RLRs), to recognize conserved molecular signatures (pathogen-associated molecular patterns, PAMPs) [1]. The RLRs, RIG-I and MDA5, function as cytoplasmic sensors, patrolling for the presence of viral RNA [2, 3]. In response to viral infection, RLRs form a complex with their essential adaptor, mitochondrial antiviral signalling protein (MAVS, also called IPS-1, VISA or Cardif) through homotypic caspase recruitment domain interaction [4–7]. The activated MAVS complex then recruits the IKK and TBK1/IKKi complexes to induce transcriptional expression of type I interferon by promoting nuclear translocation of the NF-κB and IRF3/IRF7 transcription factors, respectively, and thus elicits the innate antiviral response [8].

It has been previously demonstrated that MAVS is predominantly localized and executes its functions at the outer membrane of mitochondria, revealing that the mitochondria fundamentally provide a functional platform for innate antiviral-signalling transduction [4, 9, 10]. Recent studies have identified a number of mitochondria-associated proteins (for example, NLRX1, MFN2, TUFM and COX5B) [11–14] that interact with and negatively regulate MAVS signalling activity. These findings suggest that mechanisms involving the dynamics of mitochondrial morphology and its quality control appear to contribute to the immune balance in host cells. In addition, other emerging lines of evidence have suggested that the autophagy pathway is also engaged in controlling MAVS-mediated antiviral signalling [15]. These findings provide a potential link between mitochondrial homeostasis and MAVS-mediated antiviral signalling; however, fundamental issues of whether and how MAVS mediates mitochondrial homeostasis remain to be explored.

Autophagy is an evolutionarily conserved pathway that is responsible for maintaining cellular homeostasis by removing damaged organelles and unwanted proteins [16, 17]. This process is initiated by an isolated membrane that engulfs targeted proteins or damaged organelles and then elongates and seals to eventually form a double-membrane vesicle known as an autophagosome. Next, autophagosomes are fused with lysosomes to form autolysosomes for either bulk degradation of their contents or selective degradation of their target organelles, such as the damaged mitochondria [18, 19]. In selective autophagy, the removal of damaged organelles (for example, mitochondria) has been proposed to be regulated by several mechanisms. For example, autophagy receptors, such as ATG32, NIX and FUNDC1, have important roles in targeting autophagic cargo via interaction with LC3 for autophagosome formation and subsequently delivering the cargo to lysosomes for degradation [20–23]. Accumulating evidence revealed that the PINK1–Parkin pathway also has important roles in regulating mitochondrial homeostasis by removing the damaged mitochondria [24]. A recent study showed that PINK1 recruited autophagy receptors NDP52 and Optineurin to the mitochondria to active mitophagy in a Parkin-independent manner [25]. However, molecular mechanisms that control mitochondrial homeostasis under viral infection remain largely unknown.

In this study, we provide compelling evidence that MAVS activation is sufficient to induce the autophagic signalling that mediates the turnover of damaged mitochondria. We show that a mutant form of MAVS lacking the LC3-binding ‘YxxI’ motif fails to support an efficient interaction of MAVS with LC3, suggesting that MAVS directly interacts with LC3 through this motif. Furthermore, our experimental evidence reveals that the loss of MAVS reduced level of the type II form of LC3 that is induced by RLR signalling. Collectively, our findings support the hypothesis that MAVS acts as a novel receptor for mitochondria-associated autophagic signalling to maintain mitochondrial homeostasis.

## Results

### MAVS interacts with LC3

**Figure 1:**
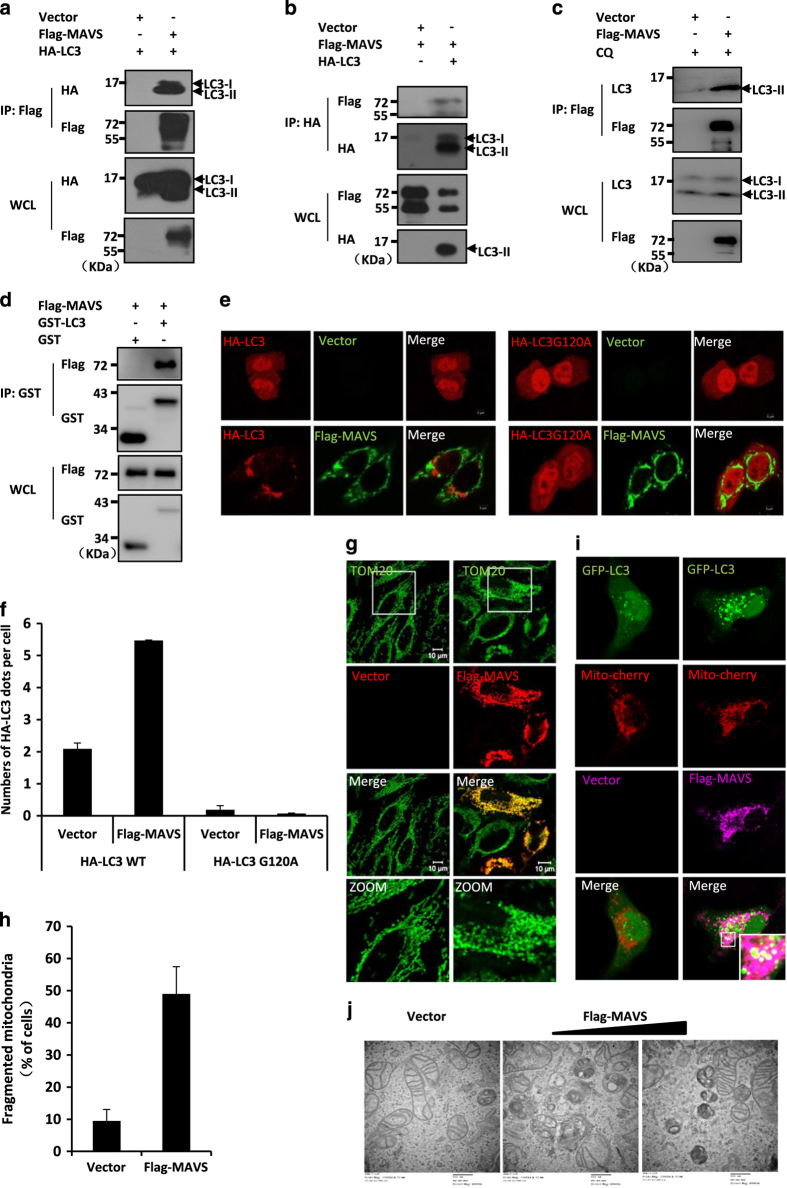
Overexpression of MAVS induces autophagy. (**a**, **b**) HEK293 cells were transfected with expression plasmids for Flag-MAVS or an empty vector together with HA-LC3 or an empty vector. Twenty-four hours after transfection, cells were lysed and immunoprecipitated with anti-Flag beads (**a**) or anti-HA beads (**b**), followed by western blot analysis with the indicated antibodies. (**c**) HEK293 cells were transfected with expression plasmids for Flag-MAVS or an empty vector. Twenty-four hours after transfection, cells were treated by chloroquine (CQ) for another 4 h. The cells were lysed and immunoprecipitated with anti-Flag beads, and then followed by western blot analysis with anti-Flag or anti-LC3 antibodies. WCL (bottom), expression of transfected or endogenous proteins in whole-cell lysates. (**d**) HEK293 cells were transfected with Flag-MAVS. Twenty-four hours after transfection, the cells were lysed. The cell lysate and purified GST-LC3 fusion protein or GST control protein were incubated with Glutathion Sepharose 4B beads for 2 h at 4 °C, then the precipitated complex was analysed by western blot assay with the indicated antibodies. (**e**) HeLa cells were transfected with Flag-MAVS (green) or an empty vector and HA-LC3 (red) or HA-LC3G120A (red) as indicated. Twenty-four hours after transfection, the cells were fixed and stained with anti-Flag or anti-HA antibodies and then imaged by confocal microscopy. (**f**) The numbers of GFP-LC3 dots were quantified in **e** (mean+s.d. of ⩾50 cells). (**g**) HeLa cells were transfected with Flag-MAVS (red) or an empty vector. Twenty-four hours after transfection, the cells were fixed and stained with anti-Flag and anti-TOM20 (green) antibodies, and then the morphologic changes of the mitochondria were observed by confocal microscopy. (**h**) The percents of cells with fragmented mitochondria were quantified in **g** (mean+s.d. of ⩾100 cells). (**i**) HeLa cells were co-transfected with Mito-Cherry (red), GFP-LC3 (green) and Flag-MAVS (purple) or an empty vector. Twenty-four hours after transfection, the cells were fixed and stained with anti-Flag antibody or mounted onto slides directly and then the colocalization of the GFP-LC3 dots and the mitochondria were imaged by confocal microscopy. (**j**) HeLa cells were transfected with increasing amounts of MAVS expression plasmids, and an empty vector was used to balance the total DNA amount. Thirty-six hours after transfection, the cells were fixed and then analysed using electron microscopy. Scale bar, 500 nm.

LC3, the mammalian homologue of yeast Atg8, is essential for the formation of autophagosomes [26]. Upon autophagic stimulation, LC3-I is converted into a phosphatidylethanolamine-conjugated form, LC3-II, which specifically interacts with the autophagy receptor, assisting in target recognition. We have previously shown that the activation of MAVS signalling increased reactive oxygen species (ROS) production and induced LC3 puncta formation when GFP-LC3, an autophagy indicator, was co-expressed with MAVS in transfected cells [14]. It has been previously shown that MAVS physically interacts with ATG5, a membrane component of the autophagosome [27]. Combing these findings, we reasoned that the activation of MAVS might function as cellular stimuli to activate the autophagic signalling pathway. Given that the overexpression of MAVS transiently induced the formation of LC3-II [14], we tested if MAVS physically interacts with LC3 by performing co-immunoprecipitation experiments. As shown in Figure 1a–c, both endogenous LC3-II and HA-tagged LC3-II were easily detected in the MAVS-immunoprecipitated complexes, whereas LC3-I exhibited a much weaker association with MAVS, suggesting that MAVS preferentially forms a complex with the phosphatidylethanolamine-conjugated form of LC3 (LC3-II), which is required for the formation of autophagosomes. Consistently, purified glutathione *S*-transferase (GST)-tagged LC3, but not the GST control, could pull down ectopically expressed Flag-MAVS from human embryonic kidney (HEK) 293 cell lysates (Figure 1d). Furthermore, we examined whether the endogenous MAVS protein could form a complex with LC3, and found that, although endogenous LC3 was weakly detected in endogenous MAVS complex in cells under a normal condition, a much stronger interaction of MAVS-LC3 was observed upon vesicular stomatitis virus (VSV) infection (Supplementary Figure S1). In line with this, LC3-II levels were significantly increased upon VSV infection. Thus, our findings provide direct biochemical evidence that links the activation of MAVS signalling to the autophagy pathway through preferentially interacting with LC3-II.

### Activation of MAVS signalling is sufficient to induce autophagy

Given that MAVS induces the formation of GFP-LC3 puncta, which is used as one of the most reliable autophagy markers [14], and preferentially interacts with LC3-II, we reasoned that the activation of MAVS might be sufficient to induce autophagic signalling. To test this idea, we first explored whether MAVS induces autophagosome formation by generating a mutant form of LC3, HA-LC3G120A, which carries a point mutation for glycine to alanine at position 120 of LC3. This point mutation blocks the conjugation of LC3 with phosphatidylethanolamine, which prevents LC3 from associating with autophagosomes [26]. As shown in immunostaining assays, the overexpression of MAVS reliably induced the dot formation of wild-type HA-LC3 in cells. By contrast, no apparent puncta signal was detected when the mutant form of LC3, HA-LC3G120A, was expressed in cells under the same conditions (Figure 1e and f), suggesting that the overexpression of MAVS is sufficient to induce autophagosome formation.

Because MAVS is a mitochondria-associated protein, we next investigated how MAVS activation affects the behaviour of mitochondria by performing immunostaining assays using the antibody of TOM20, a mitochondrial outer membrane protein. As shown in Figure 1g and h, the overexpression of MAVS in HeLa cells not only led to morphologic changes of their mitochondria but also caused significant mitochondrial fragmentation in a sub-population of the mitochondria, when compared with the control transfection with an empty vector. More interestingly, a significant portion of the GFP-LC3 puncta induced by activated MAVS was observed in close association with the fragmented mitochondria (Figure 1i). Given that MAVS is a mitochondria-associated protein, we next sought to determine whether the overexpression of other mitochondrial proteins causes mitochondrial fragmentation. As shown in Supplementary Figure S2, we overexpressed Bcl2, a mitochondrial protein, and found no apparent evidence of mitochondrial fragmentation. These results suggested that MAVS overexpression could specifically induce mitochondrial fragmentation.

Given previous observations showing that MAVS overexpression could induce apoptosis [28, 29], we then sought to ask whether morphologic changes of the mitochondria is attributed to apoptosis when MAVS was overexpressed in cells. To do so, we detected the signal of apoptosis in cells with overexpressing MAVS at various time courses. As shown in western blot assays, we found that apoptosis was detected at 48 h post MAVS overexpression, whereas fragmented mitochondria, could be induced much earlier, at 24 h post MAVS overexpression (Supplementary Figure S3; Figure 1g). These findings suggest that morphologic changes of the mitochondria induced by overexpression of MAVS are not likely due to apoptosis.

We next performed electron microscopy assays. As shown in Figure 1j, autophagic vesicles were not observed in the control cells that were transfected with an empty vector. In contrast, single-membrane autolysomes were clearly detected in the transfected cells expressing MAVS. In addition, cells that were overexpressing MAVS had a much higher ratio of the damaged mitochondria with disrupted cristae to healthy mitochondria than control cells, indicating that these mitochondria might be undergoing turnover through the autophagy pathway. Collectively, our findings suggest that ectopic expression of MAVS is sufficient to induce autophagy signalling.

### MAVS is essential for the autophagy activation mediated by the RLR pathway

**Figure 2:**
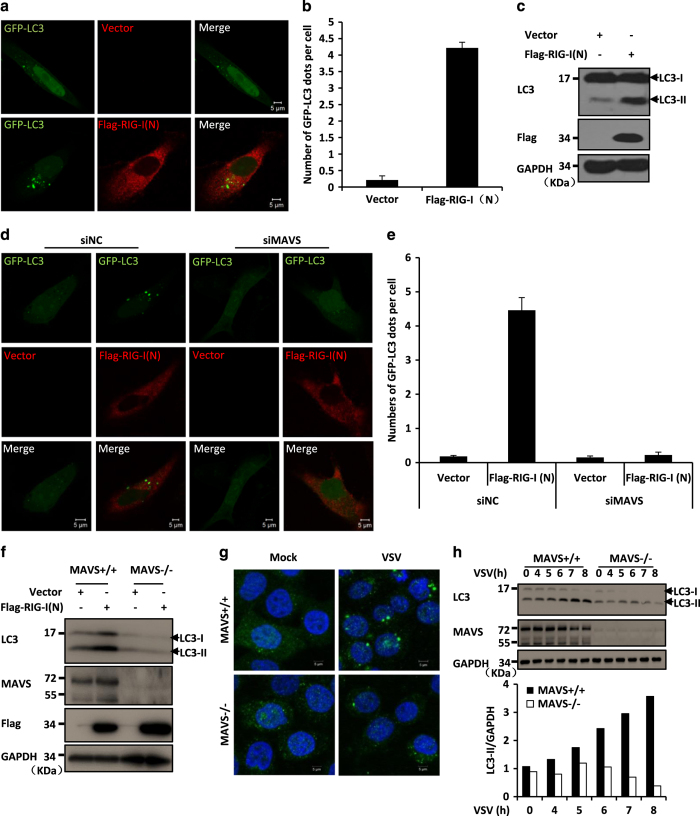
MAVS is essential for the autophagy activation mediated by the RLR pathway. (**a**) HeLa cells were transfected with GFP-LC3 (green) and Flag-RIG-I (N) (red) or an empty vector. Twenty-four hours after transfection, the cells were fixed, stained with anti-Flag antibodies or mounted onto slides directly, and imaged by confocal microscopy. (**b**) The numbers of GFP-LC3 dots were quantified in **a** (mean+s.d. of ⩾50 cells). (**c**) HeLa cells were transfected with Flag-RIG-I (N) or an empty vector. Twenty-four hours after transfection, the total protein was extracted and subjected to immunoblotting analysis with the indicated antibodies. (**d**) HeLa cells were transfected with negative control (NC) or MAVS RNAi oligos. Twenty-four hours after transfection with the oligos, GFP-LC3 and Flag-RIG-I (N) or an empty vector were transfected into the RNAi-transfected cells. Twenty-four hours after the second transfection, the cells were fixed, stained with anti-Flag antibody or mounted onto slides directly, and imaged by confocal microscopy. (**e**) The numbers of GFP-LC3 dots were quantified in **d** (mean+s.d. of ⩾50 cells). (**f**) HeLa wild-type (WT) or MAVS knockout cells, which were generated by the CRISPR/Cas9 system, were transfected with Flag-RIG-I (N) or an empty vector. Twenty-four hours after transfection, the total protein was extracted and subjected to immunoblotting analysis with the indicated antibodies. (**g**) HeLa WT or MAVS knockout cells were left untreated or infected with VSV at a multiplicity of infection (MOI) of 1 for 8 h. Cells were fixed, stained with anti-LC3 antibody (green) and 4′,6-diamidino-2-phenylindole (blue), and then imaged by confocal microscopy. (**h**) HeLa WT and MAVS knockout cells were left untreated or infected with or without VSV at MOI of 1 for the indicated times. Total protein was extracted and subjected to immunoblotting analysis with the indicated antibodies. Densitometry analyses to quantify the levels of LC3-II expression are shown in the bottom panel.

Because MAVS functions as a critical regulator in RLR-antiviral signalling, we next investigated whether the activation of RLR signalling induces autophagy. To do so, we overexpressed RIG-I (N), an activated form of RIG-I, to activate MAVS signalling in HeLa cells, as RIG-I acts upstream of MAVS in the RLR signalling pathway. As shown in Figure 2a and b, the overexpression of RIG-I (N) was sufficient to induce LC3 puncta formation in the HeLa cells expressing GFP-LC3. Western blot assays revealed that LC3-II levels were increased when cells were transfected with RIG-I (N) (Figure 2c). Similar results were obtained when cells were transfected with poly(I:C) to stimulate the RLR-mediated antiviral signalling pathway (Supplementary Figure S4a and b).

To test whether MAVS is required for RLR-mediated autophagy, we co-transfected with RIG-I (N) and GFP-LC3 in MAVS knockdown HeLa cells, and found that the knockdown of MAVS significantly reduced the RIG-I (N)-induced GFP-LC3 puncta formation when compared with the control (Figure 2d and e; Supplementary Figure S4c). To further validate this observation, we generated MAVS KO HeLa cell lines by employing the CRISPR/Cas9 system [30]. As expected, a western blot results showed that the knockout of MAVS prevented LC3-II formation in cells following RIG-I (N) overexpression (Figure 2f) or transfection with poly(I:C) (Supplementary Figure S4d). These results suggest that MAVS is essential for RLR-induced autophagic signalling.

Previous studies have shown that viral infection by VSV can induce activation of autophagic signalling [12, 27]. We next examined whether MAVS is functionally required for this process. As shown in Figure 2g and h, VSV infection led to fewer LC3-II puncta and reduced levels of LC3-II in MAVS knockout cells than in control cells, suggesting that a MAVS deficiency reduces VSV-induced autophagic signalling. We then assessed the specificity of the role of MAVS in activating RLR-mediated autophagic signalling and tested whether MAVS is also involved in regulating starvation-induced autophagy. We found that MAVS failed to regulate this type of autophagy induced by the treatment with Earle’s balanced salt solution (Supplementary Figure S5). Thus, our results suggest that MAVS has a specific role in regulating the activation of autophagy mediated by RLR signalling.

### MAVS induces autophagy through its transmembrane domain and via ROS induction

**Figure 3:**
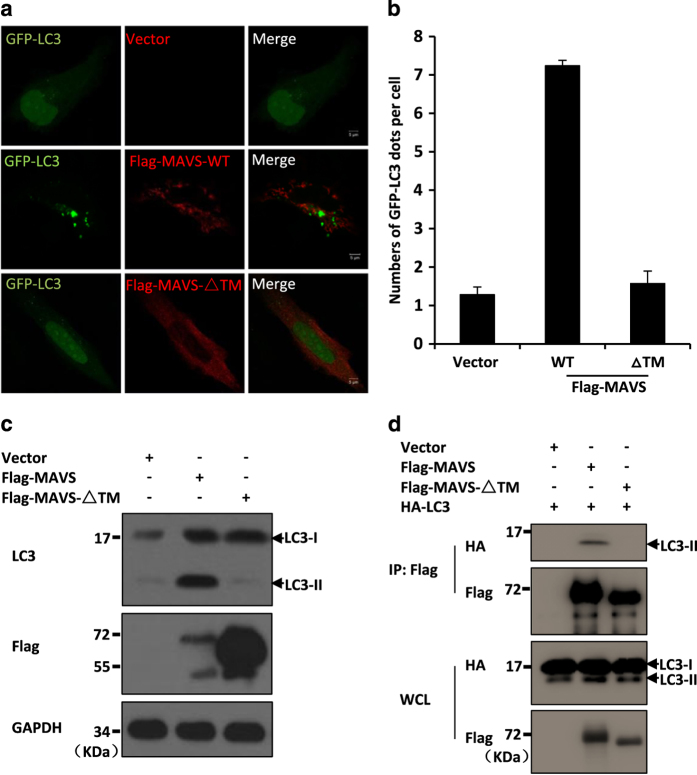
MAVS induces autophagy through its TM domain. (**a**) HeLa cells were transfected with GFP-LC3 (green) together with an empty vector, Flag-MAVS or Flag-MAVS-ΔTM vector. Twenty-four hours after transfection, the cells were fixed, stained with anti-Flag antibodies (red), and imaged by confocal microscopy. (**b**) The numbers of GFP-LC3 dots were quantified in **a** (mean±s.d. of ⩾50 cells). (**c**) HeLa cells were transfected with the expression plasmids encoding a control vector, Flag-MAVS or Flag-MAVS-ΔTM. Twenty-four hours after transfection, the total protein was extracted and subjected to immunoblotting analysis with the indicated antibodies. (**d**) HEK293 cells were transfected with the indicated expression plasmids. Twenty-four hours after transfection, the cell lysates were prepared and immunoprecipitated with anti-Flag beads then followed by western blot analyses.

We next sought to understand how MAVS activates the RLR-mediated autophagy pathway. Given that MAVS localizes to the outer membrane of mitochondria through its transmembrane (TM) domain, we investigated whether the TM domain is required for the role of MAVS in the induction of autophagy signalling. We co-expressed a mutant form of MAVS (MAVS-ΔTM), which lacks the TM domain, with GFP-LC3 in HeLa cells. As shown in Figure 3a–c, in contrast to expression of the wild-type form of MAVS, which was sufficient to induce LC3 puncta formation and increase the levels of LC3-II, expression of the truncated MAVS-ΔTM failed to either induce GFP-LC3 puncta formation or increase the LC3-II levels in cells. Consistent with these results, MAVS-ΔTM was not able to form a complex with LC3 (Figure 3d). Thus, our combined results suggest that the mitochondrial localization of MAVS is essential for it to induce autophagy.

Previous studies have demonstrated that MAVS overexpression causes a reduction of the mitochondrion membrane potential and consequently increases the levels of mitochondria-derived ROS production [14, 28]. Because ROS production is involved in regulating the autophagy pathway [31–33], we sought to test whether mitochondria-derived ROS induced by MAVS overexpression contributes to the MAVS-mediated induction of autophagy. We measured the abundance of LC3 puncta formation in cells expressing GFP-LC3 together with MAVS vector that were also treated with or without Mito-TEMPO, a scavenger specific for mitochondrial ROS. As shown in Supplementary Figure S6a, Mito-TEMPO treatment significantly suppressed the GFP-LC3 puncta formation induced by MAVS overexpression. Similar results were obtained when another ROS inhibitor, PDTC, was used in analogous assays (Supplementary Figure S6a). In agreement with these findings, the results from western blot assays show that the level of LC3-II induced by MAVS overexpression was reduced by the treatment with Mito-TEMPO or PDTC (Supplementary Figure S6b). Thus, our results suggest that the mitochondrial localization of MAVS is important for autophagy induction, and that this induction is, at least in part, mediated through the mitochondrial ROS that are induced by the activation of MAVS signalling.

### Activation of MAVS promotes mitochondrial turnover

**Figure 4:**
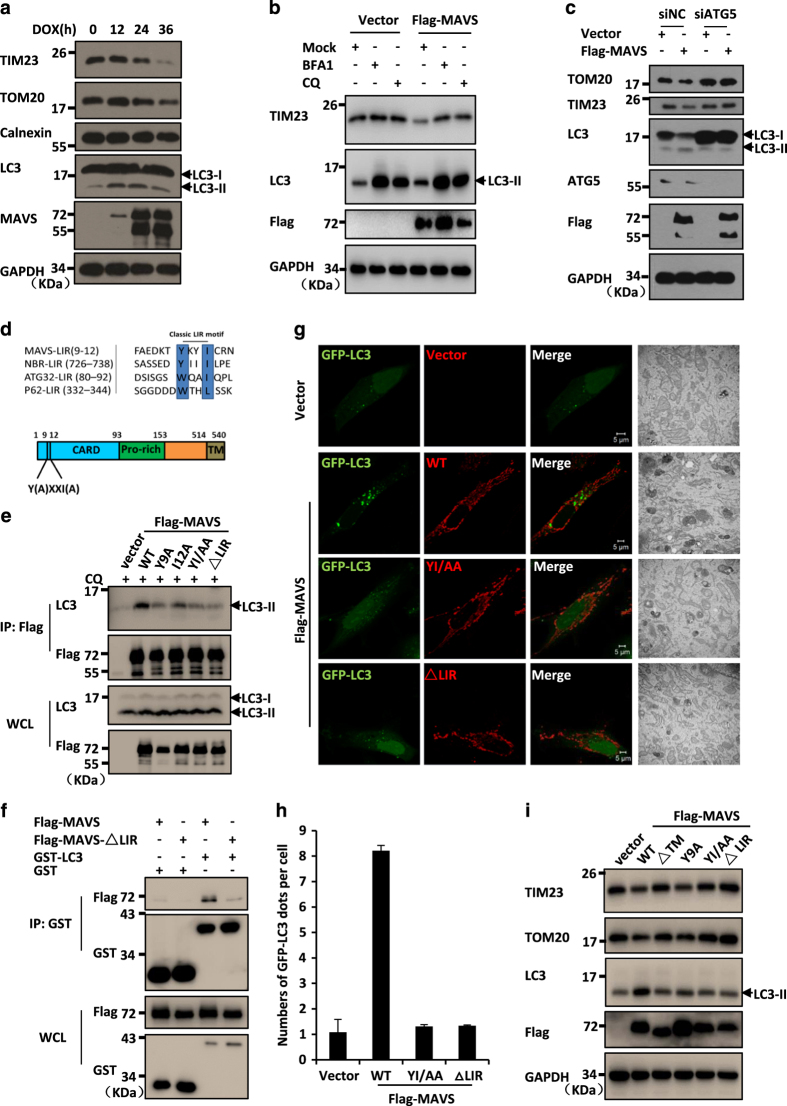
MAVS functions as a potential mitophagy receptor through interaction with LC3 via its LIR motif. (**a**) HeLa cell lines stably expressing MAVS induced by DOX were treated with DOX (0.25 μg ml^−1^) at the indicated times, then cells were lysed directly and subjected to immunoblotting analysis with the indicated antibodies. (**b**) HeLa cells were transfected with an empty vector or Flag-MAVS. Twenty-four hours after transfection, the cells were either left untreated or treated with bafilomycin A1 (BFA1) for 12 h or chloroquine (CQ) for 4 h. After treatment, the total protein was extracted and subjected to immunoblotting analyses with the indicated antibodies. (**c**) HeLa cells were transfected with negative control (NC) or ATG5 RNAi oligos. Twenty-four hours after transfection, Flag-MAVS or empty vectors were transfected into the RNAi cells. Thirty-six hours after the second transfection, the total protein was extracted and subjected to immunoblotting analysis with the indicated antibodies. (**d**) The sequences of LIR motif (W/YXXI/L) in MAVS were aligned manually with typical autophagy receptors. (**e**) HEK293 cells were transfected with expression plasmids encoding a control vector, wild-type Flag-MAVS or its LIR mutant forms. Twenty-four hours after transfection, the cells were treated with CQ for 4 h. Cell lysates were prepared and immunoprecipitated with anti-Flag beads, followed by western blot analyses. (**f**) HEK293 cells were transfected with Flag-MAVS or Flag-MAVSΔLIR. Twenty-four hours after transfection, the cell lysates and purified GST-LC3 fusion protein or GST control protein were incubated with Glutathion Sepharose 4B beads for 2 h at 4 °C, then the precipitated complex was analysed by western blot assay with the indicated antibodies. (**g**) HeLa cells were co-transfected with GFP-LC3 (green) together with an empty vector or wild-type or LIR mutant forms of MAVS (red). Twenty-four hours after transfection, the cells were fixed and imaged by confocal microscopy (left panel) or by electron microscopy (right panel). (**h**) The numbers of GFP-LC3 dots were quantified in **g** (mean±s.d. of ⩾50 cells). (**i**) HeLa cells were transfected with the indicated plasmids. Thirty-six hours after transfection, the total protein was extracted and subjected to immunoblotting analysis with the indicated antibodies.

Because MAVS acts as a mitochondrial outer membrane protein to mediate antiviral signalling and the overexpression of MAVS resulted in mitochondrial damage, we next tested whether MAVS-induced autophagy is responsible for mitochondrial quality control through the removal of damaged mitochondria. To investigate this question, we examined whether MAVS-induced autophagy targets the mitochondria for their turnover. We performed western blot assays and measured the dynamic turnover of mitochondria by examining the levels of TOM20 (for outer membrane) and TIM23 (for inner membrane) proteins in HeLa cells with inducible MAVS overexpression. As shown in Figure 4a, upon MAVS expression induced by DOX, the levels of both TOM20 and TIM23 proteins were progressively reduced in a time-dependent manner, as compared with their levels in control cells. Notably, the reduction of mitochondrial protein levels was prevented when autophagy inhibitors, bafilomycin A1 or chloroquine, were used to treat the cells, suggesting that the activation of MAVS indeed induces mitochondrial turnover, at least in part, through autophagy-lysosome-mediated degradation (Figure 4b).

To further test whether autophagy is involved in MAVS-induced mitochondrial turnover, we knocked down ATG5, a key component of autophagosomes. We found that, in contrast to control cells, the overexpression of MAVS failed to induce autophagic signalling and maintain mitochondrial homeostasis when ATG5 was knocked down in cells (Figure 4c). These findings strongly suggest that MAVS is involved in maintaining mitochondrial homeostasis via the autophagy pathway.

### MAVS acts as a potential receptor to interact with LC3 via the LIR motif

Mitophagy is a type of autophagy that selectively targets the damaged mitochondria through the interaction of mitophagy receptors with LC3 [34]. Previous studies have identified several mitochondrial outer membrane proteins, such as ATG32 and FUNDC1, that function as receptors to regulate mitophagy [20, 22, 23]. Given that MAVS is a mitochondrial outer membrane protein and that activated MAVS is sufficient to induce autophagy and interacts with LC3-II, we speculated that MAVS maintains mitochondrial homeostasis by functioning as a mitophagy receptor. It has been documented that mitophagy receptors normally contain a typical motif with a consensus sequence of W/YxxL/I, which mediates the interaction between LC3 and mitophagy receptor and the subsequent recognition of autophagosomes [35]. Interestingly, by performing a sequence alignment analysis, we observed that MAVS carries a typical motif Y(9)xxI(12) (also called the LC3-interaction region, LIR) at its N-terminal region (Figure 4d). Of note, this motif is partially overlapped with the MAVS caspase recruitment domain and is exposed to the cytosol.

To test whether the LIR motif is important for MAVS interaction with LC3, we generated a series of mutant forms of MAVS: the MAVS-Y9A and MAVS-I12A point mutants, the MAVS-YI/AA double-point mutant, and the LIR-deleted form, MAVS-ΔLIR. As shown in co-immunoprecipitation experiments, although endogenous LC3-II was clearly detected in the wild-type MAVS immunoprecipitants, the association affinity of the MAVS-LIR mutants with LC3-II was notably reduced. Particularly, we found that deletion of the LIR motif nearly abolished the MAVS-LC3-II interaction (Figure 4e). Consistently, purified GST-tagged LC3 could pull down ectopically expressed wild-type Flag-MAVS, but not Flag-MAVS-ΔLIR from HEK293 cell lysates (Figure 4f). Thus, our results suggest that the LIR motif is essential for MAVS to interact with LC3-II. Next, we employed these MAVS mutants to ask whether the LIR motif is functionally required for MAVS-induced autophagy. As shown in Figure 4g and h, in contrast to the wild-type MAVS, the MAVS-YI/AA double-point mutant and the LIR-deleted form induced significantly fewer GFP-LC3 puncta, and the associated electron microscopy experiment showed similar results. Consistent with these findings, no apparent reduction of the mitochondrial proteins TOM20 and TIM23 was detected when either of these two LIR mutants of MAVS was overexpressed in cells, indicating that these two mutants failed to maintain mitochondrial turnover (Figure 4i). These results suggest that LIR is essential for the activation of autophagic signalling and the maintenance of mitochondrial homeostasis, namely, mitophagy, by MAVS. In addition, MAVS-ΔTM also failed to maintain mitochondrial turnover, suggesting that the mitochondrial localization of MAVS is essential for it to induce mitophagy (Figure 4i). Collectively, our findings support the hypothesis that MAVS functions as a potential mitophagy receptor in regulating mitochondrial turnover when host cells are challenged by excessive antiviral signalling.

### MAVS recruits TRAF2/6 onto the mitochondria for inducing mitophagy

**Figure 5:**
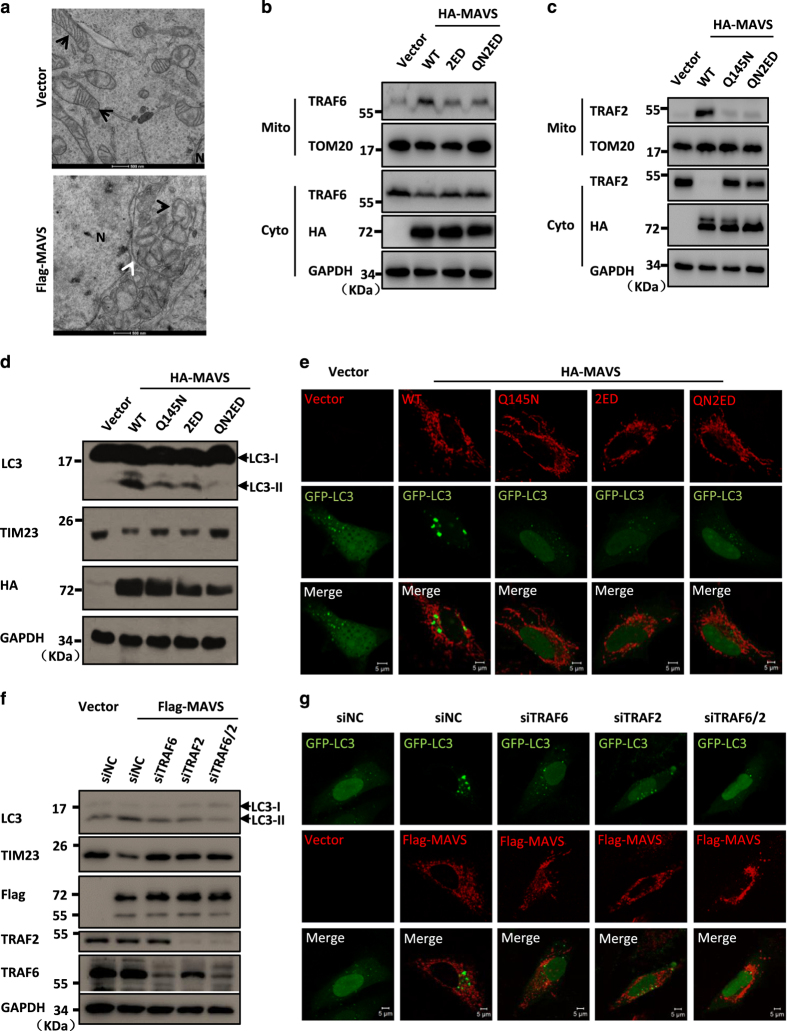
Recruitment of TRAF2 and TRAF6 onto the mitochondria induce MAVS-mediated mitophagy. (**a**) HeLa cells were transfected with Flag-MAVS or an empty vector. Twenty-four hours after transfection, the cells were fixed and analysed by electron microscopy. The black arrows indicate the mitochondria, the white arrow shows a double membrane outside of the clustered mitochondria and N indicates nucleus. (**b**, **c**) HeLa cells were transfected with the indicated plasmids. Twenty-four hours after transfection, the crude mitochondrial fraction (P5) and the cytosolic fraction (S5) were separated, and the translocation of TRAF6 (**b**) or TRAF2 (**c**) was detected by western blot analysis. (**d**) HeLa cells were transfected with the indicated plasmids. Thirty-six hours after transfection, the total protein was extracted and subjected to immunoblotting analysis with the indicated antibodies. (**e**) HeLa cells were co-transfected with GFP-LC3 (green) together with an empty vector or wild-type or TRAF2/6-binding mutant forms of MAVS (red). Twenty-four hours after transfection, the cells were fixed, stained anti-HA antibody and imaged by confocal microscopy. (**f**) HeLa cells were transfected with RNAi oligos (20 nM) targeting TRAF6 or TRAF2, or with negative control (NC) oligos as indicated. Twenty-four hours after transfection, the cells were transfected with an empty vector or Flag-MAVS. Thirty-six hours after the second transfection, the total protein was extracted and subjected to immunoblotting analysis with the indicated antibodies. (**g**) HeLa cells were transfected with RNAi oligos (20 nM) targeting TRAF6 or TRAF2, or with NC oligos as indicated. Twenty-four hours after transfection with the oligos, GFP-LC3 and Flag-MAVS or an empty vector were transfected into the RNAi-transfected cells. Twenty-four hours after the second transfection, the cells were fixed, stained with anti-Flag antibody and then imaged by confocal microscopy.

Having uncovered the role of MAVS as a mitophagy receptor in the regulation of mitochondrial turnover, we next sought to explore the molecular mechanism underlying this action. The results of an electron microscopy experiment demonstrated that the overexpression of MAVS in HeLa cells changed the normal mitochondrial distribution into the mitochondrial aggregates that were surrounded by double membranes (Figure 5a). Considering that PINK1 and Parkin have important roles in selective autophagic elimination of the damaged mitochondria in Parkinson’s disease, and that Parkin, a ubiquitin E3 ligase, can be recruited by PINK1 onto impaired mitochondria, presumably for ubiquitinating mitochondrial surface proteins [36, 37]. We therefore sought to determine whether Parkin is involved in MAVS-mediated regulation of mitochondrial turnover. Given Parkin’s translocation from the cytosol to the mitochondria is a prerequisite for Parkin’s actions on the mitochondria, we performed immunostaining experiments, and found that Parkin failed to translocate onto the mitochondria when MAVS was co-overexpressed with Parkin (Supplementary Figure S7), suggesting that Parkin is not probably required for MAVS induced mitophagy. In line with this, the overexpression of MAVS induced mitochondrial turnover in HeLa cells, which reportedly lack functional Parkin protein [38]. Thus, these findings suggest that the MAVS-induced mitophagy likely functions in parallel with the cellular process of Parkin-induced mitophagy.

MAVS harbours multiple binding sites for TRAF proteins [39], including the PVQET motif (143–147), which is responsible for TRAF2 binding and recruitment, and the PGENSE (153–158) and PEENEY (455–460) motifs, which are required for TRAF6 binding. The TRAF2/6 proteins have been shown to act as ubiquitin E3 ligases and to function, in conjugation with MAVS, in playing a crucial role in the activation of antiviral immune signalling [39]. To test whether the mitochondrial recruitment of TRAF proteins is involved in MAVS-mediated autophagy/mitophagy, we first explored the biological importance of these TRAF-binding motifs in MAVS and generated a series of mutant forms of MAVS, MAVS^Q145N^ (MAVS-Q145N), MAVS^E155D/E457D^ (MAVS-2ED) and MAVS^Q145N/E155D/E457D^ (MAVS-QN2ED). In agreement with previous findings, immunoprecipitation experiments revealed that both TRAF2 and TRAF6 proteins could be associated with the wild-type MAVS protein in transfected cells (Supplementary Figure S8). Importantly, our biochemical fraction analysis showed that overexpression of MAVS resulted in a significant mitochondrial translocation of TRAF2 and TRAF6 proteins from the cytosolic compartment (Figure 5b and c). In contrast, all mutant forms of MAVS (MAVS-Q145N, MAVS-2ED and MAVS-QN2ED) failed to recruit TRAF2 and TRAF6 proteins onto the mitochondria (Figure 5b and c). Furthermore, we found that MAVS TRAF-binding sites mutants had a significantly reduced ability to induce autophagy and to maintain mitochondrial homeostasis compared with the abilities of wild-type MAVS (Figure 5d and e). This suggests that the accumulation of TRAF proteins at the mitochondrial membrane recruited by MAVS is essential for MAVS-induced autophagy and mitophagy.

To further test the requirement of TRAF proteins for MAVS-induced autophagy, we performed RNA interference (RNAi) experiments to knock down TRAF2 and TRAF6 individually or in combination, followed by MAVS overexpression. We found that the knockdown of either TRAF2 or TRAF6 alone or the knockdown of TRAF2 and TRAF6 in combination disrupted the MAVS-induced autophagy signalling and inhibited the mitophagy process (Figure 5f and g). Thus, together our findings suggest that the recruitment of TRAF2/6 onto the mitochondria is essential for MAVS-induced mitophagy.

### MAVS aggregation is essential for its function in mediating mitophagy

**Figure 6:**
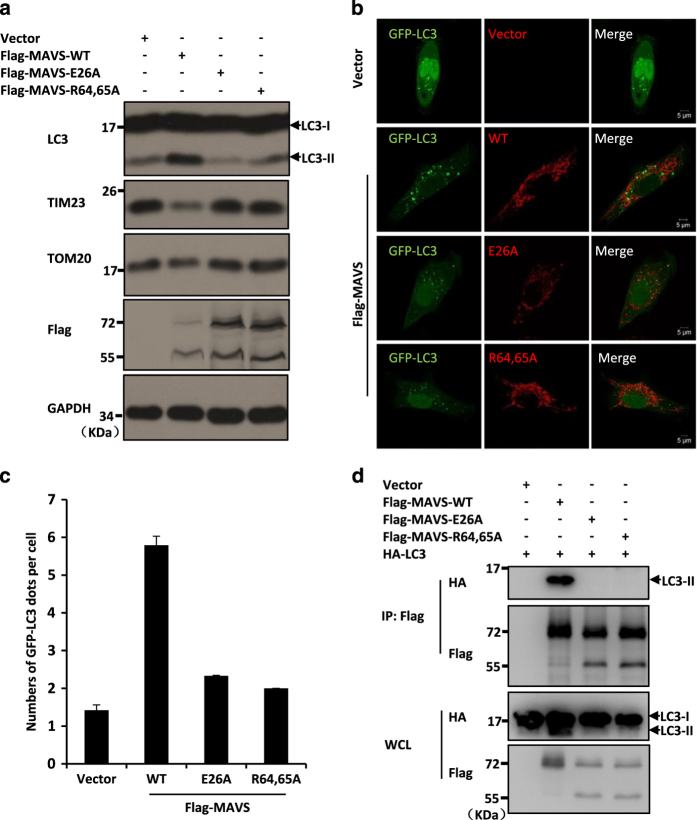
The role of MAVS aggregation in its function in mediating mitophagy. (**a**) HeLa cells were transfected with the indicated plasmids. Thirty-six hours after transfection, the total protein was extracted and subjected to immunoblotting analyses with the indicated antibodies. (**b**) HeLa cells were co-transfected with GFP-LC3 (green) together with wild-type Flag-MAVS or its aggregation mutant forms (red). Twenty-four hours after transfection, the cells were fixed, stained by the anti-Flag antibody and imaged by confocal microscopy. (**c**) The numbers of GFP-LC3 dots were quantified in (**b**) (mean±s.d. of ⩾50 cells). (**d**) HEK293 cells were transfected with the indicated plasmids. Twenty-four hours after transfection, the cells were lysed and immunoprecipitated with anti-Flag beads, and then followed by western blot analysis.

Previous studies have shown that MAVS aggregates on the mitochondrial membrane upon activation of the RLR signalling pathway [40] and that polymerization of MAVS is required for its recruitment of TRAF proteins [39]. Given that aggregation of MAVS has been shown to be negatively regulated by ATG5 [14], we sought to test whether the aggregated form of MAVS is required for MAVS to mediate mitophagy. We generated two mutant forms of MAVS, MAVS-E26A and MAVS-R64,65A, because both mutations abrogate the ability of MAVS to form aggregates on the mitochondrial membrane [39]. As shown in Figure 6a, although the expression of wild-type MAVS increased LC3-II formation and decreased the expression levels of mitochondrial membrane proteins TIM23 and TOM20, the expression of either MAVS-E26A or MAVS-R64,65A lost the ability of MAVS to enhance the level of LC3-II or to induce the turnover of TIM23 and TOM20 proteins. To confirm our findings, we next performed immunostaining experiments to test whether aggregation mutant forms of MAVS affect the GFP-LC3 distribution in cells, and as shown in Figure 6b and c, the expression of either MAVS-E26A or MAVS-R64,65A produced notably fewer GFP-LC3 puncta in cells than the expression of wild-type MAVS did.

Previous studies have shown that the MAVS-E26A and MAVS-R64,65A aggregation mutations disrupt the MAVS-TRAFs association [39]. We next tested whether these two mutations that disrupt MAVS aggregation also influence the MAVS-LC3 interaction. The results from immunoprecipitation assays show that, compared with wild-type MAVS, these MAVS aggregation mutants lost their binding affinity to LC3-II (Figure 6d). Taken together, our results strongly suggest that MAVS aggregation is essential for its role in mediating mitophagy.

## Discussion

Autophagy is an evolutionarily conserved pathway critical for maintaining cellular homeostasis by removing unwanted proteins and damaged organelles, such as dysfunctional mitochondria [16, 17]. Accumulating evidence suggest that the proper turnover of mitochondria via the selective autophagy pathway, namely, mitophagy, is important for development and tissue homeostasis [21, 41, 42]. A number of mechanisms that regulate distinct mitophagy processes have been proposed to account for mitochondrial quality control, such as PINK1/Parkin-, NIX- and FUNDC1-mediated mitophagy pathways [21, 22, 36].

Innate immunity is the first line of host defence against invading microorganisms, such as viruses. Recent studies have established a link between autophagy and the antiviral innate immune system [14, 27]. For example, ATG5 was found to regulate RLR signalling through association with MAVS and RIG-I [27], and its deficiency leads to the enhancement of RLR signalling in a ROS-dependent manner [43]. NLRX1/TUFM were also found to form complexes with MAVS and ATG5, thereby regulating the RLR signalling and autophagy [11, 12]. However, little is known about if antiviral-signalling-induced dysfunctional mitochondria are recognized and removed via autophagy and how this cellular signalling is mediated. In this study, we provide evidence showing that not only the activation of MAVS is necessary and sufficient to induce autophagy, but also that MAVS functions as a potential mitophagy receptor to mediate proper mitochondrial turnover. Thus, our study uncovers a novel mechanism by which MAVS regulates the autophagy pathway to maintain mitochondrial homeostasis.

### A novel role for MAVS in regulating the autophagy pathway

The mitochondrial outer membrane-associated protein MAVS has been shown to have critical roles in regulating antiviral-signalling transduction. Cumulative studies have suggested that the mitochondrial membrane provides an important platform for MAVS to mediate downstream antiviral signalling [4, 40]. Moreover, antiviral signalling (for example, activation of MAVS) leads to increased ROS production, morphologic changes of mitochondria and dysfunctional mitochondria that have a significant reduction of their membrane potential (loss of ΔΨm) [14, 28]. However, little is known about the mechanisms of how these phenotypes are induced. In this study, we show that the activation of MAVS-mediated antiviral signalling is sufficient to induce autophagic signalling, which is illustrated by several lines of evidence. First, the overexpression of MAVS-induced significant GFP-LC3 puncta formation, an indicator of autophagic signalling activation, and increased the levels of LC3-II, a phosphatidylethanolamine-modified form of LC3 that is regarded as a key component of autophagosomes. Second, MAVS preferentially associated with LC3-II and other component of the autophagosomes, such as ATG5 [27]. Last, the results from our immunostaining assays reveal that the overexpression of MAVS led to mitochondria fragmentation, and that a significant portion of GFP-LC3 puncta was closely associated with fragmented mitochondria, suggesting that these the mitochondria underwent turnover mediated by autophagy. In addition to MAVS overexpression, activation of RLR signalling by expression of RIG-I(N), transfection with poly(I:C) or infection with VSV also activated autophagic signalling. Interestingly, knockdown or knockout of MAVS significantly reduced RLR-induced autophagic signalling, suggesting that MAVS is critical for autophagy signalling transduction when host cells are challenged by virus. Thus, our study uncovers a novel important role for MAVS in transducing autophagy signalling, in addition to its previously described essential role in mediating antiviral signalling.

### MAVS acts as an autophagy receptor to maintain mitochondrial homeostasis

We have previously shown that the mitochondrial electron transfer machinery component COX5B functions in concert with ATG5, an autophagosome component, to negatively regulate antiviral signalling by suppressing MAVS aggregation. This led to the question of whether or not MAVS is an important target in the autophagy pathway in terms of its regulation of mitochondrial behaviour upon viral challenge. In this study, we provide compelling evidence showing that MAVS is likely a mitochondrial autophagy receptor that is specifically recognized by LC3-II. First, like other mitophagy receptors, MAVS has a typical LC3-binding motif, LIR. This motif in MAVS is essential for its interaction with LC3-II, and it likely serves as a target recognition site for autophagosomes. Second, mutations in or deletion of the LIR motif in MAVS disrupted its ability to induce autophagic signalling and to maintain mitochondrial turnover. Third, MAVS-induced autophagy and mitophagy appears to depend on mitochondrial localization, as the deletion of its mitochondrial TM domain completely abolished its capacity to induce autophagic signalling and subsequently promote mitophagy.

Given that MAVS is mitochondria-associated protein, the next important question becomes as to whether MAVS has a specific role in maintaining mitochondrial homeostasis. Several lines of evidence support this specificity of MAVS. First, MAVS, but not Bcl2 (another mitochondrial protein), could form a complex with LC3 (Supplementary Figure S9a). Second, in contrast to MAVS, the overexpression of Bcl2 failed to induce autophagy and mitophagy (Supplementary Figure S9b). Third, we generated a construct expressing a mitochondrial targeting GFP protein, GFP-MAVS^TM^, in which the TM domain of MAVS was fused with a GFP, and found that overexpression of GFP-MAVS^TM^, again failed to induce autophagy and mitophagy (Supplementary Figure S9b). These results suggest a specific feature of MAVS in maintaining mitochondrial homeostasis.

Of note, by western blotting after VSV infection, we failed to detect the changes of TOM20 and TIM23 expression. Nevertheless, we could observe colocalization of TIM23 with LC3 puncta in a small portion of mitochondria in the MAVS^+/+^ cells, when compared with that in MAVS^−/−^ cells upon VSV virus infection (Supplementary Figure S10). These findings indicate that there is probably only a small portion of damaged mitochondria that undergo mitophagy process.

Previous studies have revealed that Parkin, a ubiquitin E3 ligase, can be recruited onto the depolarized mitochondria by PINK1 to ubiquitinate mitochondrial surface proteins, which recruit adaptors for recognition by LC3-II and thereby support the mitophagy process [44]. Our results show that Parkin failed to translocate onto the mitochondria when MAVS was co-overexpressed with it, and the overexpression of MAVS is sufficient to induce mitochondrial turnover in HeLa cells, which lack endogenous Parkin. Thus, Parkin is likely not involved in MAVS-mediated mitophagy. However, our results suggest that MAVS could recruit other ubiquitin E3 ligases, such as the TRAF2/6 proteins, onto the mitochondria through its multiple TRAF-binding sites. Mutations of TRAF-binding sites and knockdown of TRAF2/6 both disrupted the ability of MAVS to induce mitophagy. Future experiments should investigate whether TRAF2/6 proteins perform a similar role in this pathway as Parkin does in its related pathway.

## Materials and Methods

### Cell culture, antibodies, reagents and virus

HEK293 cells, HeLa cells, U2OS cells and Vero cells were cultured in Dulbecco’s modified Eagle medium (Invitrogen, Carlsbad, CA, USA) supplemented with 10% fetal bovine serum (Hyclone, South Logan, UT, USA) and antibiotics (100 U ml^−1^ penicillin and 100 μg ml^−1^ streptomycin, Invitrogen). Antibodies used in this study for western blot or immunofluorescence assays were as follows: mouse anti-Flag (Sigma, St Louis, MO, USA), rabbit anti-LC3 (Novus, Littleton, CO, USA and MBL, Woburn, MA, USA), mouse anti-TIM23 (BD, Waltham, MA, USA), mouse anti-GAPDH and rabbit anti-Caspase-3 (Sungene Biotech, Tianjin, China), rabbit anti-Myc and rabbit anti-HA (MBL), rabbit anti-ATG5 (Epitomics, Cambridge, MA, USA), and rabbit anti-TOM20, mouse anti-Calnexin, mouse anti-TRAF2 and rabbit anti-TRAF6 (Santa Cruz Biotechnology, Dallas, TX, USA), rabbit anti-Cleaved Caspase-3 (Cell Signalling Technology, Danvers, MA, USA); the antibody against MAVS was produced by our lab [14]. Mito-TEMPO was purchased from Santa Cruz Biotechnology or Enzo Life Science, Farmingdale, NY, USA. Pyrrolidinedithiocarbamic acid ammonium salt (PDTC), bafilomycin A1 and chloroquine were purchased from Sigma. VSV was propagated in Vero cells.

### Plasmids, RNAi and transfection

Mammalian Flag-MAVS, Flag-RIG-I(N) and HA-LC3 overexpression plasmids were constructed by standard molecular biology techniques. MAVS mutants lacking the LIR motif or TM domain were cloned by overlap cloning techniques. Various mutants, including the MAVS LIR motif site mutants, TRAF protein-binding site mutants, aggregation mutants and HA-LC3G120A, were generated by PCR using *Pfu* Turbo DNA polymerase (Invitrogen). The plasmids of Mito-cherry and GFP-LC3 were generously provided by Dr Quan Chen. The RNAi sequences used in this study are as follows (only the sense strand is shown): negative control, GUUCUCCGAACGUGTCACGU; ATG5, GAAGUUUGUCCUUCUGCUA; MAVS, CCACCUUGAUGCCUGUGAA; TRAF6, CCACGAAGAGAUAAUGGAU; TRAF2, GAUGUGUCUGCGUAUCUAC. HeLa cells were transfected with RNAi oligos at a final concentration of 20 nM using the calcium phosphate precipitation method. The RNAi cells were transfected again with the indicated plasmids using lipofectamine 2000 (Invitrogen) or MagaTran1.0 (Origene, Rockville, MD, USA) according to its manufacturer’s instructions.

### CRISPR/Cas9-mediated MAVS knockout cell lines

Single-guide RNA for targeting human MAVS genomic locus was designed and cloned into pX330 vector [30]. HeLa cells were co-transfected with the plasmids containing each target single-guide RNA sequence and pPGKpuro, and then cultured in medium containing puromycin (2 μg ml^−1^) for 2 days for selection. After 2 weeks, single colonies were selected, and MAVS expression was tested by immunoblotting. The sequences targeting MAVS were (5′–3′): TCTGACCTCCAGCGGGCATC and GACTCCAGGGGGCCACCATC.

### Immunofluorescent confocal microscopy

HeLa cells were grown on gelatin-coated glass coverslips and transfected with the indicated plasmids by the calcium phosphate precipitation method. Twenty-four hours after transfection, the cells were washed with phosphate-buffered saline (PBS), fixed in 4% paraformaldehyde in PBS for 10 min at room temperature, washed three times with PBS, permeabilized and blocked with 0.2% TritonX-100 in PBS containing 5% bovine serum albumin for 30 min. Cells were then incubated with the primary antibody, then with the secondary antibody and finally imaged by a Zeiss LSM 710 META (Zeiss, Oberkochen, Germany) laser scanning confocal system.

### Co-immunoprecipitation and immunoblot analyses

HEK293 cells were washed with PBS, lysed in 0.5% TritonX-100 lysis buffer (50 mM Tris–Cl at pH 7.4, 150 mM NaCl, 0.5% TritonX-100, 10% glycerol and 1 mM EDTA) containing protease inhibitors on ice for 30 min, and sonicated briefly. Then, the supernatants were incubated with anti-Flag (Sigma), anti-Myc (Selleckchem) or anti-HA (MBL) agarose beads for 4–6 h at 4 °C. The beads were washed three times with lysis buffer and subjected to immunoblot analysis with the indicated antibodies. For semi-endogenous and endogenous co-immunoprecipitation, cells were lysed with radioimmunoprecipitation assay lysis buffer (50 mM Tris–Cl at pH 7.4, 150 mM NaCl, 1% NP-40, 1% sodium deoxycholate, 0.1% SDS, 10% glycerol and 1 mM EDTA). Immunoblotting was carried out by standard procedures.

### Electron microscopy

HeLa cells were transfected or treated as indicated. Cells were fixed in 2.5% glutaraldehyde in 0.1 M PBS (pH 7.4) overnight at 4 °C. After being washed with PBS, the samples were post-fixed with 1% osmium tetroxide containing 0.8% potassium ferricyanide at room temperature for 1 h, embedded in Spurr’s resin, sectioned, doubly stained with uranyl acetate and lead citrate, and analysed using a Zeiss EM 10 transmission electron microscope (Zeiss, Oberkochen, Germany).

### Subcellular fractionation

Cells were washed with PBS, resuspended in hypotonic buffer (20 mM HEPES–KOH (pH 7.4), 10 mM KCl, 1.5 mM MgCl_2_, 1 mM EDTA and 1 mM EGTA) containing a protease inhibitor cocktail (Roche, South San Francisco, CA, USA), and homogenized by douncing 15 times with a dounce homogenizer. Then, the same volume of 0.5 M d-mannose was added to the samples and the homogenate was centrifuged at 500* g* for 5 min. The resulting supernatant was then centrifuged at 5 000 *g* for 10 min. The pellet (P5) contained crude mitochondria, and the supernatant contained the cytosolic fraction (S5). The fractions were lysed and analysed by western blot assays.

### Purification of GST-LC3 and *in vitro* pull-down assay

Full-length LC3 was cloned into the pGEX-4 T-1 vector and expressed in *E. coli* (BL21). The fusion protein was purified by Glutathion Sepharose^TM^ 4B beads (GE) from the bacteria cell lysate. For the *In vitro* pull-down assay, HEK293 cells were transfected with Flag-MAVS or its mutants, and lysed in 0.5% TritonX-100 lysate buffer at 24 h post transfection. Cell lysate and GST-LC3 fusion protein or GST control protein were incubated with Glutathion Sepharose^TM^ 4B beads for 2 h at 4 °C, and the beads were washed three times with cell lysis buffer, then the precipitated complex was analysed by western blot assays.
