# Supplementary material for: MAVS maintains mitochondrial homeostasis via autophagy
Source: Cell Discov. 2016 Aug 16;2:16024–. doi: 10.1038/celldisc.2016.24 (PMC4986202; doi:10.1038/celldisc.2016.24)
Supplement: Supplementary Figure S1 [file celldisc201624-s1.pdf]

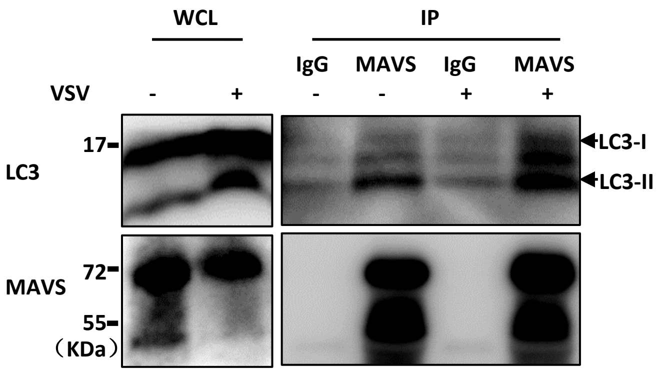

Figure S1. The interaction between endogenous MAVS and LC3 is enhanced by VSV infection

HEK293 cells were left untreated or infected with VSV for 6 hours, then the cells were lysed by RIPA buffer and incubated with anti-MAVS antibody or control IgG, then immunoprecipitated with proteinA/G beads , and followed by western blot analysis.
