# Supplementary material for: MAVS maintains mitochondrial homeostasis via autophagy
Source: Cell Discov. 2016 Aug 16;2:16024–. doi: 10.1038/celldisc.2016.24 (PMC4986202; doi:10.1038/celldisc.2016.24)
Supplement: Supplementary Figure S2 [file celldisc201624-s2.pdf]

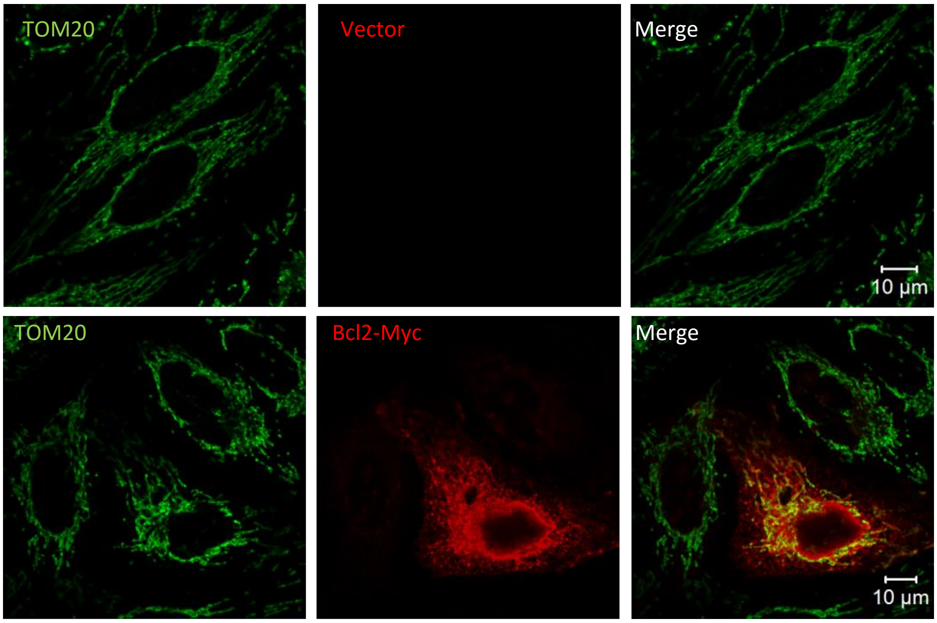

Figure S2. Overexpression of Bcl2 fails to induce mitochondrial fragmentation
HeLa cells were transfected with Bcl2-Myc or an empty vector. Twenty-four hours after transfection, the cells were fixed, stained by anti-Myc (red) or anti-TOM20 (green) antibodies, and imaged by confocal microscopy.
