# Supplementary material for: MAVS maintains mitochondrial homeostasis via autophagy
Source: Cell Discov. 2016 Aug 16;2:16024–. doi: 10.1038/celldisc.2016.24 (PMC4986202; doi:10.1038/celldisc.2016.24)
Supplement: Supplementary Figure S3 [file celldisc201624-s3.pdf]

Figure S3

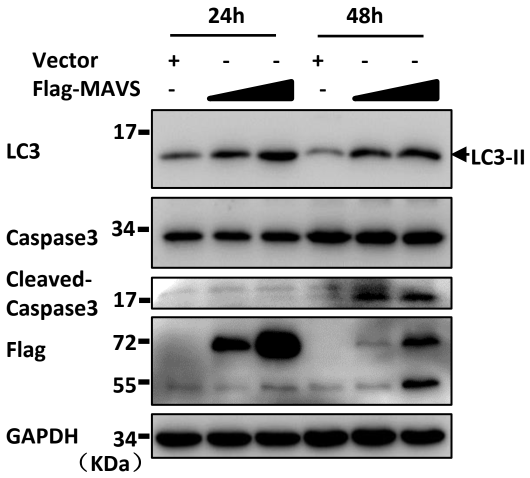

Figure S3. The effects of overexpression of MAVS in inducing apoptosis at different time points

HeLa cells were transfected with Flag-MAVS or an empty vector. Twenty-four or forty-eight hours after transfection, the total protein was extracted and subjected to immunoblotting analysis with the indicated antibodies.
