# Supplementary material for: MAVS maintains mitochondrial homeostasis via autophagy
Source: Cell Discov. 2016 Aug 16;2:16024–. doi: 10.1038/celldisc.2016.24 (PMC4986202; doi:10.1038/celldisc.2016.24)
Supplement: Supplementary Figure S4 [file celldisc201624-s4.pdf]

Figure S4

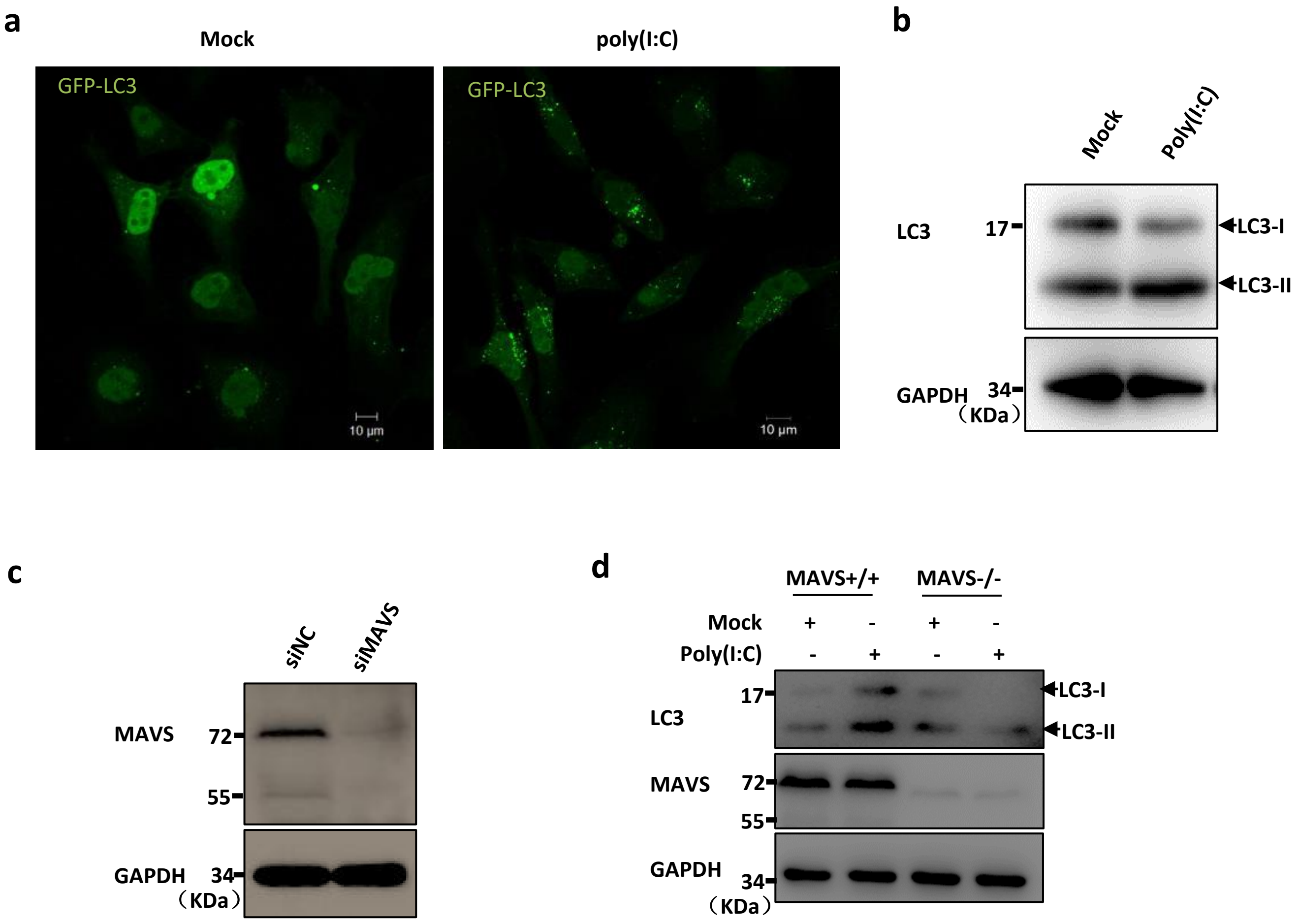

Figure S4. MAVS is essential for the autophagy activation induced by poly(I:C)

(a) HeLa cells were transfected with GFP-LC3. Twenty-four hours after transfection, the cells were transfected with poly(I:C). Twelve hours after this second transfection, the cells were fixed and then directly imaged by confocal microscopy.

(b) HeLa cells were transfected with poly(I:C). Twelve hours after transfection, the total protein was extracted and subjected to immunoblotting analysis with the indicated antibodies.

(c) HeLa cells were transfected with negative control (NC) or MAVS RNAi oligos. Thirty-six hours after transfection, the cells were lysed and subjected to immunoblotting analyses with the indicated antibodies.

(d) HeLa wild-type (WT) or MAVS knockout cells were transfected with poly(I:C). Twelve hours after transfection, the total protein was extracted and subjected to immunoblotting analysis with the indicated antibodies.
