# Supplementary material for: MAVS maintains mitochondrial homeostasis via autophagy
Source: Cell Discov. 2016 Aug 16;2:16024–. doi: 10.1038/celldisc.2016.24 (PMC4986202; doi:10.1038/celldisc.2016.24)
Supplement: Supplementary Figure S5 [file celldisc201624-s5.pdf]

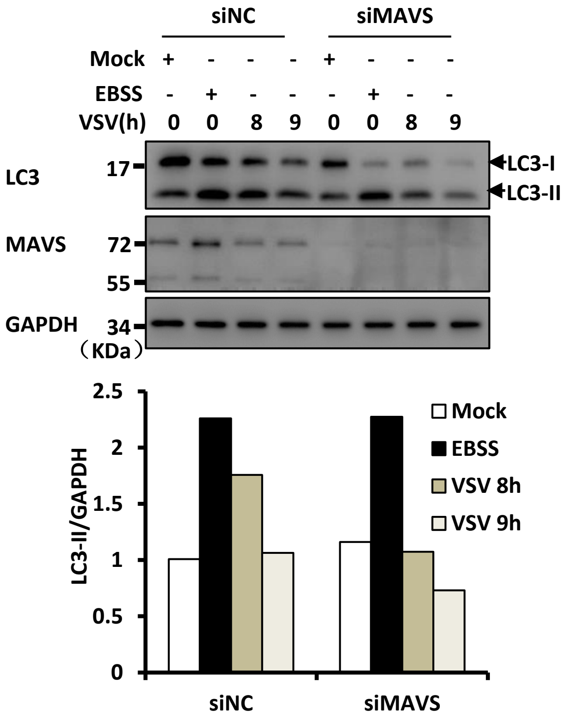

Figure S5. MAVS isn't involved in regulating the starvation-induced autophagy

U2OS cells were transfected with negative control (NC) or MAVS RNAi oligos. Thirty-six hours after transfection, the cells were left untreated, treated with Earle's Balanced Salt Solution (EBSS), or infected with VSV as indicated, and then the cells were lysed and subjected to immunoblotting analyses with the indicated antibodies. Densitometry analyses to quantify the levels of LC3-II expression are shown in the bottom panel.
