# Supplementary material for: MAVS maintains mitochondrial homeostasis via autophagy
Source: Cell Discov. 2016 Aug 16;2:16024–. doi: 10.1038/celldisc.2016.24 (PMC4986202; doi:10.1038/celldisc.2016.24)
Supplement: Supplementary Figure S6 [file celldisc201624-s6.pdf]

Figure S6

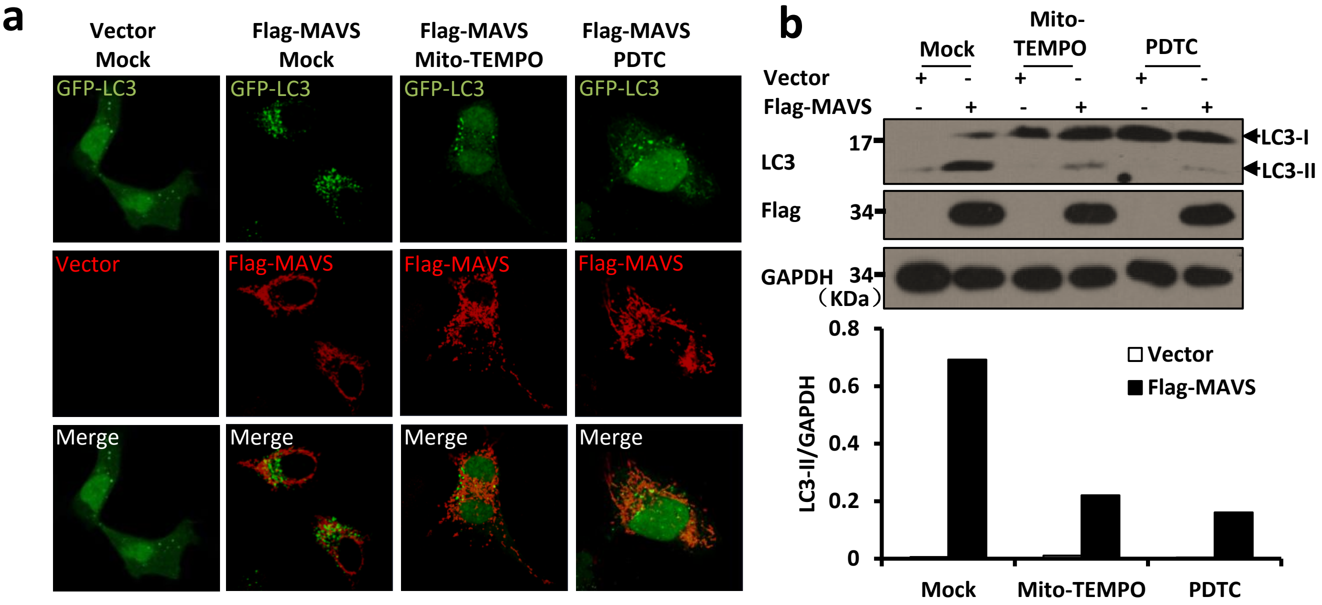

Figure S6. ROS inhibitors prevent MAVS-induced autophagy

(a) HeLa cells were transfected with GFP-LC3 (green) together with empty vector or Flag-MAVS (red) for 6 hours and then left untreated or treated with Mito-TEMPO (200 μM) or PDTC (20 μM) for 24 hours. Cells were fixed and stained with the indicated antibodies, then imaged by confocal microscopy.

(b) HeLa cells were transfected with empty vector or Flag-MAVS for 6 hours and then left untreated or treated with Mito-TEMPO (200 μM) or PDTC (20 μM) for 24 hours, then the cells were lysed directly, and subjected to immunoblotting analysis with the indicated antibodies. Densitometry analyses to quantify levels of LC3-II expression are shown in the bottom panel.
