# Supplementary material for: MAVS maintains mitochondrial homeostasis via autophagy
Source: Cell Discov. 2016 Aug 16;2:16024–. doi: 10.1038/celldisc.2016.24 (PMC4986202; doi:10.1038/celldisc.2016.24)
Supplement: Supplementary Figure S7 [file celldisc201624-s7.pdf]

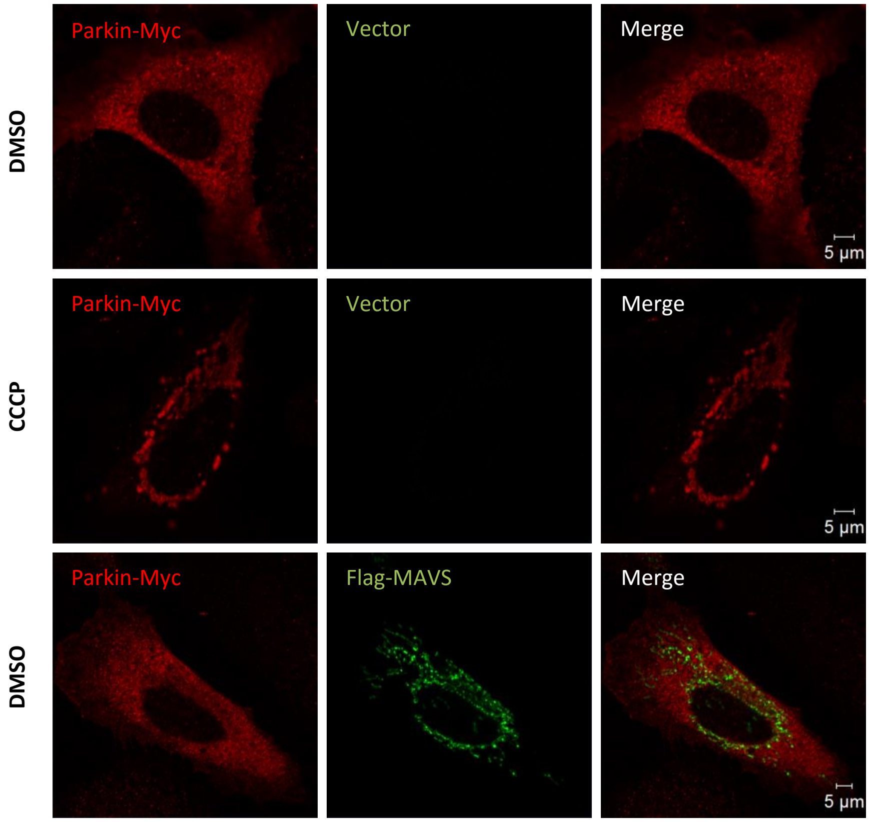

Figure S7. Parkin isn't likely involved MAVS-mediated autophagy

HeLa cells were transfected with Parkin-Myc and Flag-MAVS or an empty vector. Twenty-four hours after transfection, the cells were treated with 10μM CCCP (positive control) for 1 hours. Cells were fixed, stained by anti-Myc (red) or anti-Flag (green) antibodies, and imaged by confocal microscopy.
