# Supplementary material for: MAVS maintains mitochondrial homeostasis via autophagy
Source: Cell Discov. 2016 Aug 16;2:16024–. doi: 10.1038/celldisc.2016.24 (PMC4986202; doi:10.1038/celldisc.2016.24)
Supplement: Supplementary Figure S8 [file celldisc201624-s8.pdf]

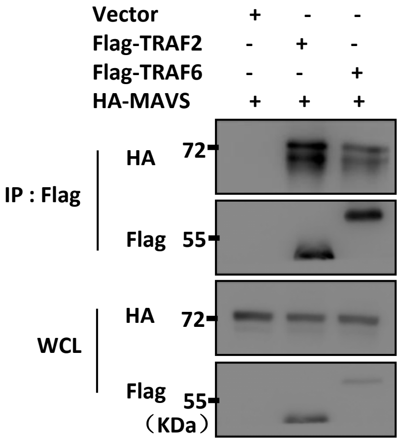

Figure S8. MAVS interacts with TRAF6/2.

HEK293 cells were transfected with the indicated plasmids. Twenty-four hours after transfection, the cell lysates were prepared and immunoprecipitated with anti-Flag beads, and followed by western blot analysis.
