# Supplementary material for: MAVS maintains mitochondrial homeostasis via autophagy
Source: Cell Discov. 2016 Aug 16;2:16024–. doi: 10.1038/celldisc.2016.24 (PMC4986202; doi:10.1038/celldisc.2016.24)
Supplement: Supplementary Figure S9 [file celldisc201624-s9.pdf]

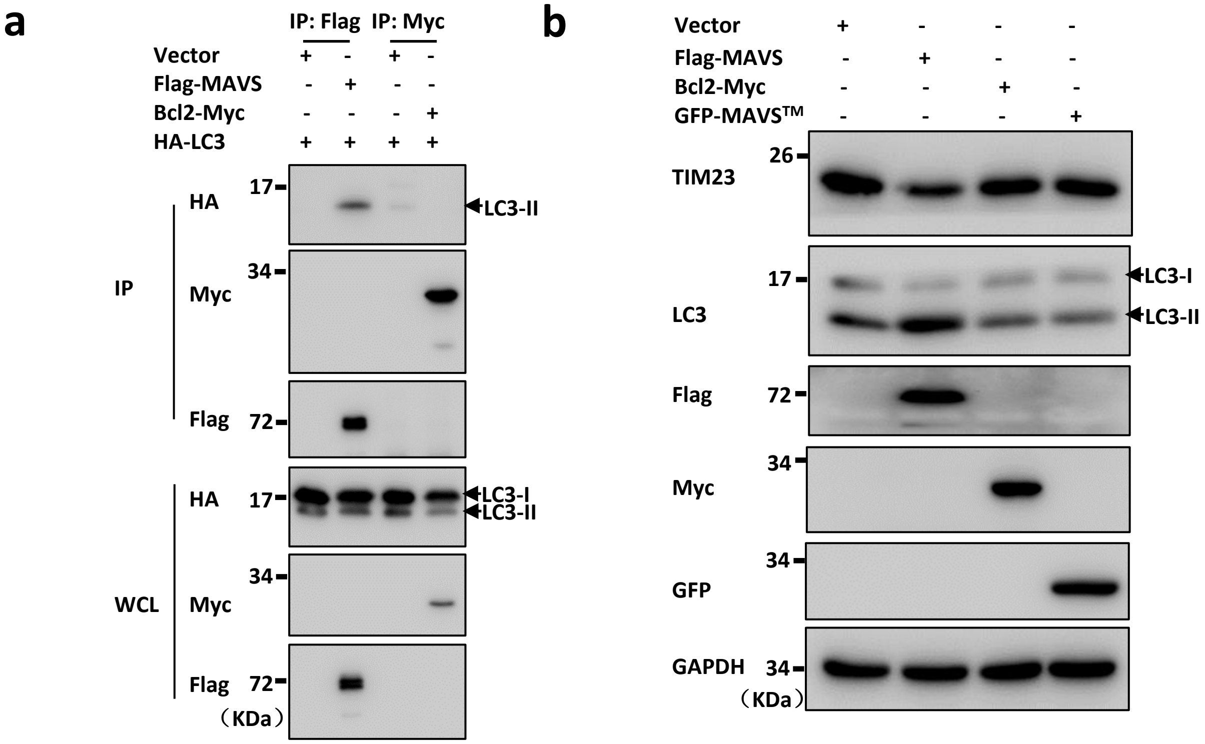

Figure S9. Overexpression of MAVS specifically induces autophagy and mitophagy

(a) HEK293 cells were transfected with the indicated plasmids. Twenty-four hours after transfection, the cell lysates were prepared and immunoprecipitated with anti-Flag beads or anti-Myc beads, and followed by western blot analysis.

(b) HeLa cells were transfected with Flag-MAVS, Bcl2-Myc, GFP-MAVS^{TM} or an empty vector. Thirty-six hours after transfection, the total protein was extracted and subjected to immunoblotting analysis with the indicated antibodies.
