# Supplementary material for: MAVS maintains mitochondrial homeostasis via autophagy
Source: Cell Discov. 2016 Aug 16;2:16024–. doi: 10.1038/celldisc.2016.24 (PMC4986202; doi:10.1038/celldisc.2016.24)
Supplement: Supplementary Figure S10 [file celldisc201624-s10.pdf]

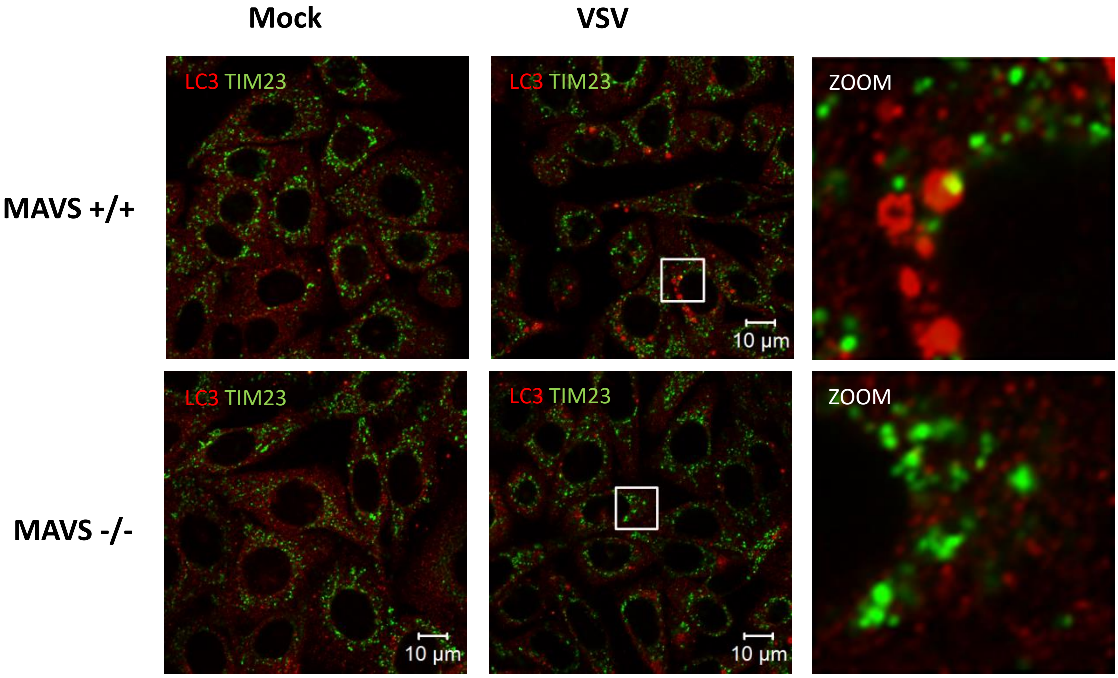


**Figure S10. Colocalization of LC3 and TIM23 in WT and MAVS KO cells upon VSV infection**

HeLa WT or MAVS knockout cells were left untreated or infected with VSV at a multiplicity of infection (MOI) of 1 for 6 hours. Cells were fixed, stained with anti-LC3 antibodies (red) and TIM23 (green), and then imaged by confocal microscopy.
